# Vitamin E Requirement in Practical Feed for Farmed Atlantic Salmon (*Salmo salar*) During Smoltification and Its Long‐Term Impact on Fish Welfare

**DOI:** 10.1155/anu/8100107

**Published:** 2026-11-01

**Authors:** Pauline Wischhusen, Kristin Hamre, Per Gunnar Fjelldal, Kaja H. Skjærven, Sofie C. Remø, Katerina Kousoulaki, Anne-Catrin Adam, Ingunn Stubhaug, Antony Jesu Prabhu Philip, Nini H. Sissener

**Affiliations:** ^1^ Research Group of Feed and Nutrition Institute of Marine Research Bergen 5005 Norway imr.no; ^2^ Hamre Aquatic Nutrition Breiviken 8 Bergen 5042 Norway; ^3^ Reproduction and Developmental Biology Group Institute of Marine Research Matre Research Station Matredal 5984 Norway imr.no; ^4^ Nutrition and Feed Technology Department Norwegian Institute of Food, Fisheries and Aquaculture Research (Nofima) Bergen 5141 Norway nofima.no; ^5^ Skretting Aquaculture Innovation P.O. Box 48 Stavanger 4001 Norway skretting.com

**Keywords:** Atlantic salmon, requirement, smoltification, tocopherol, vitamin E, welfare

## Abstract

This study aimed to establish the vitamin E requirement for Atlantic salmon during smoltification and to evaluate its long‐term implications for fish welfare. Using a regression design, seven diets with graded levels of *α*‐tocopherol (aTOH) ranging from 38 to 710 mg/kg were tested in duplicate or triplicate tanks stocked with 110 Atlantic salmon (initial weight: 81 ± 2 g), respectively. After 11 weeks, 12 fish were sampled as freshwater smolts (FW‐smolts), while the remaining fish were transferred to seawater and subjected to a challenge involving combined hypoxia and temperature stress before being sampled (seawater smolt [SW‐smolt]). Additionally, 22 fish from each tank were transferred to a single sea cage and fed a common commercial diet for 5 months to assess the long‐term impact of vitamin E nutrition during smoltification on key welfare indicators in post‐smolt. No significant effects of dietary aTOH levels on growth performance were observed, but feed intake increased in FW‐smolt fed up to 82 mg/kg. A high deposition of aTOH in liver tissue was observed in both FW‐ and SW‐smolt fed more than 400 mg aTOH/kg. Malondialdehyde (MDA) levels in the liver decreased with aTOH supplementation with breakpoints at 457 mg/kg for FW‐smolt and 99 mg/kg for SW‐smolt, suggesting life‐stage‐specific differences in oxidative stress reduction. In SW‐smolt, muscle astaxanthin levels increased linearly in response to dietary aTOH. Hematocrit and hemoglobin levels were unaffected in FW‐smolt but displayed breakpoints in SW‐smolt at 367 and 450 mg/kg, respectively. Welfare assessments revealed that fish fed 38 mg/kg during smoltification had a higher incidence of cataracts at the SW‐smolt stage and complete vertebral fusion at the post‐smolt stage. These findings suggest a minimum dietary aTOH requirement of 82 mg/kg to mitigate welfare issues, while tissue homeostasis, blood marks, and MDA suggest a practical requirement around 367–450 mg/kg.

## 1. Introduction

Vitamin E encompasses a group of four tocopherols and four tocotrienols [1, 2]. The most abundant form in animals is *α*‐tocopherol (aTOH), which is known to effectively protect cell membranes and other lipids from lipid peroxidation by acting as a fat‐soluble chain‐breaking antioxidant. The vitamin E requirement has been previously established in various fish species, including Atlantic salmon (*Salmo salar*), ranging from 30 to 60 mg/kg in first‐feeding fry [3] and 120 mg/kg in fry [4], as reflected in the NRC recommendation [5]. More recently, the practical aTOH requirements were suggested to be higher in parr and post‐smolt Atlantic salmon, ranging between 150 and 310 mg/kg [6]. The vitamin E requirement in Atlantic salmon during smoltification remains unknown. Smoltification is considered to be a highly energy‐demanding period in the life cycle that prepares for the fish’s migration to the ocean by increasing their salinity tolerance [7–9]. Atlantic salmon smolt were shown to accumulate aTOH in the liver tissue, while the levels in other organs such as the kidney and viscera decrease [10]. This mobilization of vitamin E during smoltification, especially in the kidney, might be linked to osmoregulatory adaptation but likely also reflects a change in demand due to an increased metabolic rate. Smoltification is largely hormone‐driven by insulin‐like growth factor I, cortisol, and growth hormone [7, 11]. Atlantic salmon injected with growth hormone was shown to experience oxidative stress, accompanied by a decrease of liver antioxidant levels, including vitamin E [12]. This suggests that even a marginal deficient vitamin E status during smoltification could contribute to an imbalance in the cellular antioxidant system, posing a substantial threat to the robustness of fish during seawater transfer, where the fish is exposed to a variety of environmental stressors threatening the long‐term welfare [13].

Previous studies have indicated that the estimated vitamin E requirements vary depending on the selected response criterion [14]. Traditional biomarkers used to establish vitamin E requirements in fish include growth performance, tissue homeostasis, blood parameters, and oxidative stress markers [3, 15–18]. However, the dietary levels necessary to achieve optimal growth seem to be lower than those indicated by immune biomarkers [19, 20]. Further, it has been suggested that the vitamin E requirement in Atlantic salmon may increase under stressful environmental conditions [21]. Therefore, a more comprehensive understanding of the vitamin E levels needed to achieve optimal fish welfare is essential, especially during critical life stages such as smoltification, when fish experience significant metabolic and environmental changes.

A reevaluation of the vitamin E requirement in Atlantic salmon might also be warranted in view of changing diet formulations [22]. Specifically, dietary antioxidants work synergistically to protect cells from oxidative damage, and in the past, the vitamin E requirement in Atlantic salmon at the fry stage was obtained by manipulating aTOH levels as a single ingredient [3, 4]. In contrast, the much higher aTOH requirements in Atlantic salmon at the parr and post‐smolt stage were estimated from nutrient package design experiments involving the simultaneous manipulation of various micronutrient levels in the feed [6]. Vitamin E is well‐known to interact with both vitamin C and selenium [23–25]. Thereby, vitamin C was shown to protect against vitamin E deficiency in a dose‐dependent manner, possibly related to synergistic mechanisms in the protection against tissue oxidation. However, vitamin C also plays a role in vitamin E homeostasis by regenerating vitamin E radicals [26]. In addition, supplemental selenium was shown to increase hepatic tocopherol activity in fish, while a double deficiency quickly results in detrimental symptoms including anemia, muscular dystrophy, and increased mortality [23]. Due to changes in legislation, the use of synthetic antioxidants is decreasing in commercial feeds, driving a high inclusion of aTOH in both feeds and feed ingredients as a natural alternative, yielding diet formulations possibly containing excess vitamin E [27].

Therefore, this study aimed to establish practical dietary vitamin E requirements in Atlantic salmon during smoltification using feed formulated with ingredients common in commercial diets today. To evaluate vitamin E requirements under challenging conditions, the fish were additionally exposed to mild hypoxia and elevated temperature immediately after seawater transfer. Together, these factors were applied to simulate environmental stress as expected during transfer to the sea. Following, the long‐term effect of feeding graded levels of dietary vitamin E during smoltification was monitored for an additional 5 months in a sea cage while fed a common commercial diet.

## 2. Material and Methods

### 2.1. Ethical Statement

The experiment was performed according to the Norwegian laws and regulations concerning experiments with animals for research purposes. Norway has fully implemented EU Directive 2010/63/EU in regional law. Prior to the beginning of the trial, authorization was obtained from the Norwegian Food Safety Authority (Mattilsynet, ID 30333) for the planned activities.

### 2.2. Experimental Diets

The basal feed mix was formulated with ingredients used in modern commercial Atlantic salmon smolt feeds (Table 1). The 3 mm extruded pellets were produced by Skretting Aquaculture Innovation’s pilot feed plant (Stavanger, Norway). The protein and lipid contents of the diets were 44% and 20% according to proximate composition analysis (Section 2.7 for description of analytical procedure). The level of vegetable oil with 4% was below that used in commercial smolt feed today but was chosen to limit the introduction of tocopherols and tocotrienols from raw materials.

**Table 1:** Formulation and composition of experimental diets.

Ingredients (%)	Basal mix
Fish meal^a^	35
Sunflower meal^b^	10
Soy protein conc.^c^	10
Faba bean dehulled^d^	7
Wheat^e^	7
Wheat gluten^f^	10
Fish oil^g^	13
Rapeseed oil^h^	4
Vitamin premix (no aTOH)^i^	0.2
Mineral premix^j^	0.2
Yttrium premix^k^	0.1
Astaxanthin^l^	0.05
Other supplements^m^	0.5

Proximate composition	Feed, analyzed

Dry weight (%)	92
Lipid (% DM)	20
Protein (% DM)	44
Selenium (mg/kg DM)	1.0
Vitamin C (mg/kg DM)	399
TBARS (nmol/g DM)	37

^a^North Atlantic, Pelagia, Bergen, Norway.

^b^Bunge, St Louis, USA.

^c^Köster Marine Proteins Gmbh, Germany.

^d^Soufflet Negoce by Invivo, France.

^e^Lantmännen Ek För, Sweden.

^f^Beneo‐Orafti SA, Belgium.

^g^North Atlantic, Pelagia, Bergen, Norway.

^h^European Commodity Company S.a., Luxembourg.

^i^Per kg feed: 10 mg vitamin A and K3; 0.12 mg vitamin D3; 7 mg vitamin B1; 12 mg vitamin B6 and B2; 0.2 mg vitamin B12; 60 mg Ca‐D, pantotenat; 66 mg Niacin; 0.6 mg biotin; 400 mg vitamin C, Trouw Nutrition, Nutreco, Boxmeer, the Netherlands.

^j^Per kg feed: 2.5 mg Mg; 250 mg Fe; 3 mg I; 180 mg Zn; 40 mg Mn; 10 mg Cu; 0.2 mg Se as SeMet, Trouw Nutrition, Nutreco, Boxmeer, the Netherlands.

^k^Skretting AI, Norway.

^l^NHU, Zhejiand, China.

^m^Lutavit C Aquastab 35%, Trouw Nutrition, Nutreco, Boxmeer, the Netherlands; Vit B4 Choline Cl70%, Trouw Nutrition, Nutreco, Boxmeer, the Netherlands; Zinc chelate 2.5%, Trouw Nutrition, Nutreco, Boxmeer, the Netherlands.

Seven experimental diets were formulated on the basal feed mix by supplementation of graded levels of synthetic dl‐*α*‐tocopheryl acetate (Lutavit E50%, conc. 500 000 mg/kg, BASF, Germany). The estimated dietary aTOH levels ranged from assumed suboptimal at 42 mg/kg to over‐supplementation at 800 mg/kg, all within the range observed in commercial feeds in Norway [28]. The analyzed aTOH concentration in the nonsupplemented basal feed was 38 mg/kg (VE38). The aTOH concentrations in the supplemented diets were slightly lower than expected, ranging from 82% to 89% of the target concentration: 82 mg/kg (VE82), 171 mg/kg (VE171), 260 mg/kg (VE260), 350 mg/kg (VE350), 530 mg/kg (VE530), and 710 mg/kg (VE710), respectively (Table 2). Selenium and vitamin C levels were analyzed in the experimental feeds at 1.0 and 399 mg/kg, respectively. The TBARS levels averaged 37 nmol/g, giving no indication of oxidation in any of the test diets (Table 1).

**Table 2:** Target and analyzed vitamin E concentrations (mg/kg ww) of the experimental diets.

Vitamin E	VE38	VE82	VE171	VE260	VE350	VE530	VE710
*α*‐Tocopherol, target	42	100	200	300	400	600	800
*α*‐Tocopherol	38	82	171	260	350	530	710
*β*‐Tocopherol	1.6	1.3	1.6	1.6	1.5	1.8	1.4
*γ*‐Tocopherol	28	28	28	28	28	28	29
*δ*‐Tocopherol	3.0	2.7	2.8	2.7	2.8	3.0	2.8
*α*‐Tocotrienol	0.57	0.76	0.35	1.3	2.0	2.4	3.8
*β*‐Tocotrienol	11.2	10.5	10.9	10.9	10.2	10.5	11.1
*γ*‐Tocotrienol	<0.08	<0.08	<0.08	<0.08	<0.08	<0.08	<0.08
*δ*‐Tocotrienol	<0.04	<0.04	<0.04	<0.04	<0.04	<0.04	<0.04

### 2.3. Fish Husbandry and Feeding During Tank Rearing

The feeding trial was conducted at Matre Research Station, the Norwegian Institute of Marine Research’s experimental fish facility. Sixteen 360 L (0.95 × 0.95 m, 0.4 m water depth) tanks in a flow‐through system were used in this trial, corresponding to duplicate tanks per treatment, except for VE38 and VE710, which were run in triplicate to reduce uncertainty in the regression models (Figure 1). Each tank was stocked with 110 Atlantic salmon at an initial body weight of 81 ± 2 g. Prior to the beginning of the trail, the fish were acclimatized to the environmental conditions for 2 weeks. The fish were fed ad libitum over the whole duration of the trial, while uneaten feed was collected during rearing in the flow‐through system at the freshwater and seawater phases to estimate feed intake. Smoltification was initiated after 6 weeks by changing the light regime from a 12 h light: 12 h darkness cycle to continuous light.

**Figure 1:**
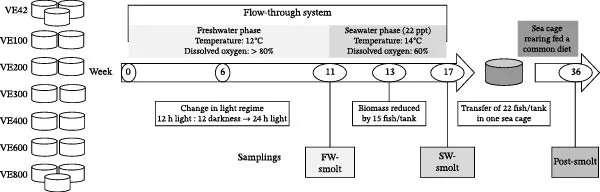
Experimental design.

Fish were sampled under freshwater conditions (12°C, dissolved oxygen: >80%) after 11 weeks of feeding as freshwater smolt (FW‐smolt). During week 12, the salinity level in the tanks was gradually increased to 22 ppt to create saltwater conditions for the remaining fish (Figure 1). Simultaneously, challenging environmental conditions were simulated by increasing the water temperature to 14°C and lowering the dissolved oxygen levels in all experimental tanks to 60%, creating mild hypoxia during seawater rearing. In week 13, a total of 15 fish were removed from each tank, respectively, to correct for optimal stocking density. The sampling of the seawater smolt (SW‐smolt) in tanks was done in trial week 18, as described in Section 2.5.

### 2.4. Sea Cage Rearing

To establish the long‐term effects of feeding graded levels of aTOH in Atlantic salmon during smoltification, 22 of the remaining SW‐smolt from each tank were pooled in a single 5 × 5 m sea cage (Figure 1). The fish transferred to the sea cage were randomly selected at the beginning of the feeding trial and had undergone additional treatment of pit‐tagging (week 2) and vaccination (week 6). The fish in the sea cage were fed a commercial diet for 5 months prior to sampling as post‐smolt.

### 2.5. Sampling Procedure

At the FW‐smolt and SW‐smolt samplings, 12 fish per tank were euthanized with an overdose of tricaine methanesulphonate (MS‐222, PharmaQ, Bergen, Norway), respectively. The fish were individually measured for weight and length, scored for external welfare parameters, and opened for nephrocalcinosis (NC) scoring (Section 2.6). Eight fish per tank were kept for whole‐body analysis. Radiographs were taken of FW‐smolt collected for whole‐body analysis as described in Section 2.6. Individual blood samples were collected using heparinized syringes from four fish per tank for hematocrit and hemoglobin analyses. After blood sampling, the fish were dissected for heart, liver, and muscle tissue. The weight of viscera, liver, and heart was recorded to calculate the somatic index of organ weights (SI = organ weight/fish body weight × 100). The liver and muscle samples of individual fish were divided into subsamples according to the planned analysis of malondialdehyde (MDA) and GSH/GSSG. In addition, pooled liver samples of the four fish were created for the determination of vitamin E, vitamin C, and selenium concentration, respectively. The tissue samples were flash frozen in liquid nitrogen at sampling and subsequently stored at −80°C until further analysis. At post‐smolt sampling, a minimum of 40 fish per dietary treatment were measured for total weight and body length. Following, the fish were evaluated for external standard welfare indicators, and radiographs were taken from all available fish to evaluate vertebral deformities. Additionally, fish from the dietary groups VE38, VE260, and VE710 (*n* = 12–14 per diet) were dissected to weigh the viscera, liver, heart, and gonads, and the sex of these fish was noted down.

### 2.6. Welfare Scoring

The welfare scoring of fish for first impression, scale loss, damage to gill operculum, and fin status was done according to the LAKSVEL protocol [29]. NC was scored following Hjelde [30] using a macroscopic scoring system. Cataract was scored in both lenses under darkened conditions using a Heine slit lamp (HEINE HSL 150 hand‐held slit lamp, HEINE Optotechnik). Indicating the degree of opacification, each lens was then given a score of 0–4, resulting in a total score of 0–8 per fish [31]. In post‐smolts, cataracts could only be scored macroscopically using the LAKSVEL protocol because no slit lamp was available. Whole fish were radiographed with a Direct Radiology System (Canon CXDI410C Wireless, CANON INC., Kawasaki, Japan) using a portable X‐ray unit (Portable X‐ray Unit Hiray Plus, Model Porta 100 HF, JOB Corporation, Yokohama, Japan) at an 88 cm distance with 40 kV and 4 mAs. Each fish was evaluated for different types of vertebral deformities as described by Witten et al. [32], and if present, the type and location were recorded. Atlantic salmon with more than 10 deformed vertebrae may have reduced growth, which is a strong indicator of reduced welfare [33].

### 2.7. Nutrient Analysis

The feed samples and whole fish were homogenized and analyzed for proximate composition. The dry matter was determined after 24 h in the oven at 105°C. Ash content was measured after combustion in the muffle oven for 16 h at 550°C. Ethyl acetate and acid extraction were used to determine lipid content in feed and fish, respectively. Protein was measured with a nitrogen analyzer as N^∗^6.25 according to AOAC official methods. Selenium was analyzed in the feed by ICP‐MS. Tocopherols and vitamin C levels were analyzed in feed and tissue by HPLC, as described in [34, 35].

### 2.8. Biochemical Tissue Analysis and Measurement of Blood Markers

MDA in the liver was analyzed as described in [36]. Reduced and oxidized glutathione were measured spectrophotometrically using the Cuvette Assay kit for GSH/GSSG (Oxford Biomedical Research, US, Product Number GT35) according to the manufacturer’s instructions. For hematocrit measurement, whole blood was immediately centrifuged in a Hettrich centrifuge using microhematocrit tubes (Am‐Heparinized, 80 iu/mL, REF162613, Vitrex Medical, Denmark). A CELL‐DYN hematology analyzer (Abbot Laboratories, Illinois, USA) calibrated with Para 12 Control blood (Streck, La Vista, NE, USA) was used to measure hemoglobin according to the manufacturer’s instructions.

### 2.9. Statistical Analysis

All statistical analyses were done using statistical software R (R Core Team 2023). All data was plotted to evaluate the distribution of the response variable in relation to the dietary vitamin E level. The data are presented as the mean and pooled standard deviation across groups calculated using the following formula with sd_*k*_ representing the standard deviation of a treatment and *n*_*k*_ representing the sample size of a treatment:

SDpooled=n1−1sd12+n2−1sd22+…+nk−1sdk2/n1+n2+…+nk−k .

Further, linear regression, polynomial, and broken‐line models were fitted to the data. Model selection was done using the AIC and *R*‐square criteria to find the best model fit. All model selection parameters are detailed in Tables S1–S3. Broken‐line models were tested for one or more breakpoints, and their 95% confidence intervals (CIs) were determined using the functions provided by the segmented package (Version 2.1‐0). Linear and polynomial models were plotted using the ggpmisc package (Version 0.6.0) as an extension to ggplot2 [37]. The welfare score data were converted from categorical to binomial prior to the statistical analysis. Following, a logistic regression with a binomial link was used to evaluate the effect of dietary aTOH level on welfare indicators.

## 3. Results

### 3.1. Fish Performance

Minor fish loss was observed in the experiment, with survival rates above 99% across all dietary treatments. The dietary aTOH level had no significant effect on the body weight or specific growth rate of FW‐smolt (Table 3). However, broken‐line regression indicates that the feed intake increased in FW‐smolt‐fed diets up to an aTOH level of 82 mg/kg (CI 63–101).

**Table 3:** Biometric measures of fish fed graded levels of *α*‐tocopherol.

	VE38	VE82	VE171	VE260	VE350	VE530	VE710	Pooled SD	*p*‐Value			Model fit/BP
									LR	P2	BL	
IBW	82.4	84.7	82.2	82.9	78.9	81.3	83.4	1.45	—	—	—	ns

*FW-smolt*												

BW	221	226	194	208	215	207	214	57	0.58	0.44	0.64	ns
K	1.18	1.19	1.15	1.22	1.18	1.17	1.19	0.13	0.95	0.99	0.83	ns
DWG	2.16	2.14	1.75	1.93	2.21	1.98	2.00	0.21	0.62	0.77	0.68	ns
SGR	1.26	1.26	1.10	1.18	1.29	1.20	1.20	0.08	0.61	0.78	0.68	ns
TGC	1.81	1.81	1.54	1.67	1.82	1.69	1.71	0.13	0.59	0.66	0.67	ns
FCR	0.68	0.81	0.98	0.88	0.79	0.91	0.84	0.10	0.29	0.16	0.17	ns
FI	1.21	1.46	1.41	1.41	1.38	1.46	1.38	0.05	0.18	0.05	<0.01	BP 82^a^; CI 63–101

*SW-smolt*												

BW	331	334	330	335	341	324	329	90	0.46	0.60	0.68	ns
K	1.21	1.20	1.22	1.21	1.21	1.22	1.19	0.10	0.24	0.08	0.21	ns
DWG	1.17	1.13	1.62	1.43	1.36	1.33	1.28	0.27	0.78	0.38	0.58	ns
SGR	0.94	0.91	1.23	1.11	1.07	1.05	1.01	0.17	0.78	0.35	0.57	ns
TGC	1.45	1.41	1.86	1.70	1.65	1.59	1.54	0.26	0.82	0.35	0.58	ns
FCR	0.90	0.83	0.59	0.77	0.71	0.82	0.82	0.16	0.94	0.36	0.55	ns
FI	2.23	1.99	1.83	2.08	2.00	2.22	2.11	0.22	0.71	0.67	0.24	ns

Post‐smolt												

BW	1070	1089	1083	1141	1079	1132	1072	374	0.91	0.72	0.64	ns
K	1.10	1.09	1.13	1.17	1.12	1.12	1.10	0.16	0.93	0.19	0.31	ns
SGR	0.72	0.74	0.75	0.77	0.69	0.73	0.74	0.30	0.99	0.99	0.67	ns

*Note:* Mean for BW and K: FW‐smolt had *n* = 36 for VE38 and VE710, *n* = 24 for others; post‐smolt had *n* = 51 for VE38, *n* = 34 for VE82, VE350, and VE530, *n* = 31 for VE171 and VE260, and *n* = 55 for VE710. For DWG, SGR, FCR, and FI: FW‐ and SW‐smolt had *n* = 3 for VE38 and VE710, *n* = 2 for VE82, VE171, VE260, VE350, and VE530; post‐smolt SGR had *n* = 22 for VE38 and VE710, and *n* = 44 for others. IBW and BW (g); FI (g/day). K = 100 x (weight/length^3^); DWG (%); SGR =  (ln(final weight)  − ln(initial weight))/days  ×  100; TGC  =  (final weight^1/3^  − initial weight^1/3^ )/*Σ*(temperature*×*days) × 1000; FCR = total feed consumed/(final body weight  − initial body weight).

Abbreviations: BL, broken‐line model; BP, breakpoint; BW, final body weight; CI, 95% confidence interval; DWG, daily weight gain; FCR, feed conversion ratio; FI, feed intake; IBW, initial body weight; K, condition factor; LR, linear regression model; ns, not significant.; P2, 2nd‐degree polynomial model; SGR, specific growth rate; TGC, thermal growth rate.

^a^aTOH ≤ 82: *y* = 1.010796 + 0.0051154*x*; aTOH ≥ *y* = 1.005384 ‐ 0.000066*x*.

Similarly, dietary aTOH level had no significant effect on growth or final body weight of SW‐smolt. The feed conversion ratio and feed intake were similar between groups for SW‐smolt. After the transfer to sea cages, the nutritional history of fish had no significant effect on the final body weight or condition factor when post‐smolt were fed a common commercial diet for 5 months (Table 3).

### 3.2. Welfare Indicators

With 98%, almost all FW‐smolt had minor fin damage, but only 7% of the fish scored above 1 without a significant effect of the dietary treatment (Table 4). In addition, 47% of the FW‐smolt showed scale loss without any significant difference between the dietary groups. On average, 86% of the FW‐smolt had cataracts, with 32% showing severe cataract scores above four, however, there were no significant differences between groups. The prevalence of NC was not significantly affected by the dietary aTOH level and was at 11% across all FW‐smolt. The prevalence of vertebral deformities in FW‐smolts ranged from 6.25% to 25% across dietary groups, with no significant influence of diet on the occurrence of various types of deformities (Table 5).

**Table 4:** Percentage of fish receiving downgrading across the LAKSVEL welfare categories first impression, damage to gill operculum, fin damage, and cataract as well as nephrocalcinosis (NC) scoring, when fed graded levels of *α*‐tocopherol.

Welfare indicator	VE38	VE82	VE171	VE260	VE350	VE530	VE710	*p*‐Value
FW‐smolt								

First impression (>0)	66	79	96	63	88	88	86	0.08
Scale loss (>0)	39	46	46	33	54	63	50	0.16
Gill damage (>0)	3	4	4	4	0	13	11	0.16
Fin damage (>0)	92	100	100	100	100	96	100	0.24
Fin damage (>1)	8	4	0	13	13	4	8	0.75
Cataract (>0)	31	54	33	54	67	42	44	0.28
Cataract (>4)	0	0	0	4	4	0	0	0.41
NC (>0)	14	8	8	8	0	29	11	0.07

SW‐smolt								

First impression	97	100	100	100	100	100	97	0.73
Scale loss	24	15	30	24	14	14	25	0.94
Gill damage	6	5	0	0	0	10	6	0.49
Fin damage (>0)	100	95	95	100	100	100	97	0.96
Fin damage (>1)	6	15	10	24	14	5	28	0.07
Cataract (>0)	92	71	83	88	88	88	92	0.03
Cataract (>4)	50	21	21	29	33	29	42	0.03
NC (>0)	17	0	8	4	17	13	17	0.68

Post‐smolt								

Scale loss (>0)	96	100	90	100	97	100	96	0.75
Gill damage (>0)	25	41	29	35	29	24	27	0.46
Fin damage (>0)	96	100	100	97	97	100	98	0.70
Fin damage (>1)	12	21	19	16	21	18	16	0.84
Cataract (>0)^a^	51	62	48	58	50	38	62	0.80

*Note:* Statistical analysis for FW‐smolt, SW‐smolt, and post‐smolt performed by logistic regression with binomial link. Superscript letters indicate a significant effect with FW‐ and SW‐smolt *n* = 36 fish for VE38 and VE710 and *n* = 24 for others and post‐smolt *n* = 51 fish for VE38, *n* = 34 fish for VE82, VE350, and VE530, *n* = 31 fish for VE171 and VE260 and *n* = 55 fish for VE710.

^a^Cataract scoring of FW‐smolt and SW‐smolt with slit lamp, but macroscopic in post‐smolt.

**Table 5:** Prevalence of vertebral deformities (%) in FW‐smolt and post‐smolt fed a diet supplemented with graded levels of *α*‐tocopherol during smoltification and then a common commercial diet for 5 months prior to sampling.

Vertebra deformity	VE38	VE82	VE171	VE260	VE350	VE530	VE710	*p*‐Value
FW‐smolt								

Any type	8.33	6.25	12.50	12.50	18.75	6.25	25.00	0.11
>10	4.17	0.00	0.00	0.00	0.00	0.00	4.17	0.73
Type 1	4.17	0.00	0.00	0.00	0.00	0.00	0.00	0.99
Type 2	4.17	0.00	0.00	0.00	0.00	0.00	4.17	0.73
Type 3	4.17	6.25	0.00	0.00	0.00	0.00	4.17	0.79
Type 4	0.00	0.00	0.00	0.00	0.00	0.00	0.00	0.99
Type 5	4.17	6.25	6.25	12.50	12.50	6.25	16.67	0.17
Type 6	0.00	0.00	6.25	12.50	0.00	6.25	8.33	0.21
Type 7	0.00	0.00	0.00	0.00	0.00	0.00	0.00	NA
Type 8	0.00	0.00	0.00	0.00	0.00	0.00	0.00	NA
Type 12	0.00	0.00	6.25	0.00	0.00	0.00	0.00	0.58
Type 13	0.00	0.00	6.25	0.00	6.25	0.00	0.00	0.74

Post‐smolt								

Any type	25.45	19.44	10.26	20.51	25.00	17.50	12.70	0.20
>10	3.64	2.78	0.00	2.56	5.00	2.50	1.59	0.71
Type 1	0.00	0.00	0.00	0.00	0.00	0.00	0.00	NA
Type 2	5.45	5.56	2.56	0.00	5.00	2.50	0.00	0.10
Type 3	1.82	0.00	0.00	0.00	0.00	0.00	0.00	0.99
Type 4	0.00	0.00	0.00	0.00	0.00	0.00	0.00	NA
Type 5	16.36	13.89	2.56	15.38	17.50	12.50	3.17	0.08
Type 6	9.09	13.89	5.13	10.26	10.00	5.00	7.94	0.50
Type 7	9.09	5.56	0.00	2.56	2.50	0.00	1.56	0.05
Type 8	1.82	0.00	0.00	5.13	0.00	5.00	0.00	0.98
Type 12	5.45	5.56	2.56	0.00	0.00	0.00	1.59	0.11
Type 13	1.82	0.00	0.00	0.00	0.00	2.50	0.00	0.81

*Note:* Statistical analysis performed by logistic regression with binomial link. The deformity types 9, 10, and 11 were not recorded in any fish. FW‐smolt had *n* = 24 fish per treatment for VE38 and VE710 and *n* = 16 fish for the other treatments, while post‐smolt had *n* = 55 for VE38, *n* = 36 for VE82, *n* = 39 for VE171, *n* = 39 for VE260, *n* = 40 for VE350, *n* = 39 for VE530, and *n* = 63 for VE710.

Major welfare issues in SW‐smolt included, similar to FW‐smolt, minor fin damage (98% of the fish) and scale loss (21% of the fish) without any significant difference between dietary treatments (Table 4). In SW‐smolt, the prevalence of cataract increased in all dietary groups compared to FW and was highest in fish‐fed VE38 and VE710 and lower at intermediate levels. A similar pattern was observed for severe cataract, which was highest in the group fed the lowest aTOH levels and less prevalent in the groups supplemented with medium levels of aTOH.

No long‐term effect of nutritional history could be detected on the standard welfare indicators in post‐smolt (Table 4). In all groups, the occurrence of vertebra deformities ranged 10%–25% of the fish. Most deformities could be considered minor, with less than 10 vertebrae affected. The deformity type 7 “complete vertebral fusion” (Figure 2) was higher in post‐smolt fed VE38 during smoltification compared to those fed higher levels of aTOH.

**Figure 2:**
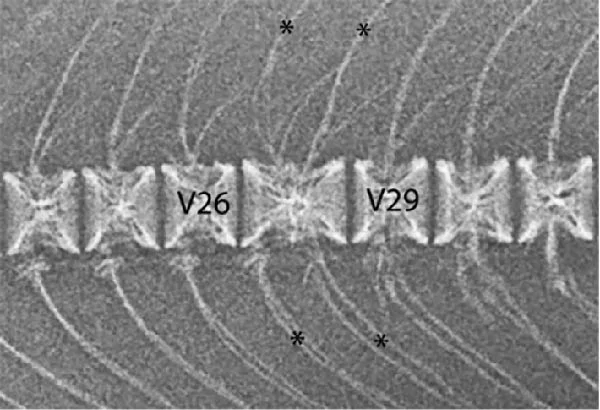
Radiographic example of a post‐smolt with a complete fusion between vertebra numbers 27 and 28. Neural and hemal arches indicated with a black asterisk.

### 3.3. Tissue Nutrient Homeostasis

Dietary aTOH levels had no significant impact on dry matter, energy, or protein content of FW‐smolt (Table 6). The VSI of FW‐smolt showed a significant decrease with increasing aTOH levels, while the hepatosomatic index (HSI) remained stable with increasing dietary aTOH supply. Broken‐line regression indicates that the cardiosomatic index (CSI) in FW‐smolts shows a linear increase up to a dietary level of 530 mg aTOH/kg (CI 372–688).

**Table 6:** Proximate composition and organ indexes of fish fed graded levels of *α*‐tocopherol.

Variable	VE38	VE82	VE171	VE260	VE350	VE530	VE710	Pooled SD	*p*‐Value			Model fit/BP
									LR	P2	BL	
*FW-smolt*												

DM	32.5	32.4	31.2	31.9	32.2	31.8	31.9	0.6	0.40	0.45	0.60	ns
Energy	9.09	9.05	8.71	9.00	9.09	8.94	8.95	0.23	0.67	0.87	0.75	ns
Lipid	13.4	13.3	13.0	14.3	13.1	13.3	13.2	0.2	0.76	0.74	0.56	ns
Protein	17.3	17.5	16.8	17.5	17.0	17.5	17.0	0.5	0.68	0.91	0.87	ns
VSI	7.6	8.0	7.7	7.6	6.2	7.1	6.7	1.3	0.02	0.05	0.18	*y* = 7.8–0.00167*x*
HSI	0.94	1.00	1.00	0.99	0.81	0.99	0.99	0.21	0.82	0.71	0.52	ns
CSI	0.13	0.14	0.14	0.14	0.14	0.16	0.14	0.01	0.11	0.04	0.05	BP530^a^; CI 372–688

*SW-smolt*												

DM	31.2	31.1	30.9	31.4	30.9	31.1	30.7	0.6	0.29	0.52	0.91	ns
Energy	8.97	8.68	8.80	8.79	8.77	8.79	8.62	0.23	0.42	0.56	0.92	ns
Lipid	12.3	12.0	12.1	12.8	12.5	12.3	11.9	0.4	0.36	0.16	0.24	ns
Protein	17.7	18.0	18.0	17.0	17.5	17.5	17.3	0.5	0.20	0.39	0.55	ns
VSI	9.6	10	12	9.0	8.9	8.8	8.5	3.5	0.17	0.39	0.75	ns
HSI	0.85	0.81	0.80	0.85	0.70	0.79	0.87	0.11	0.81	0.05	0.03	BP396^b^; CI 168–623
CSI	0.13	0.12	0.14	0.14	0.14	0.14	0.13	0.03	0.54	0.44	0.40	ns

*Note:* Mean with *n* = 3 pooled samples for VE38 and VE710 and *n* = 2 pooled samples for VE82, VE171, VE260, VE350, and VE530. Organ indexes with *n* = 12 individual fish per treatment for VE38 and VE710 and *n* = 8 individual fish per treatment for other treatments. DM (%); lipid and protein (%); energy (J/mg).

Abbreviations: BL, broken‐line model; BP, breakpoint; CI, 95% confidence interval; CSI, cardiosomatic index; DM, dry matter; HSI, hepatosomatic index; LR, linear regression model; ns, not significant; P2, 2nd‐degree polynomial model; VSI, viscerasomatic index.

^a^aTOH ≤ 530: *y* = 0.1324 + 3.793 × 10^-5^ *x*; aTOH ≥ 530: *y* = 0.191919 ‐ 7.437 × 10^-5^ *x*.

^b^aTOH ≤ 396: *y* = 0.8623329 ‐ 0.000329*x*; aTOH ≥ 396: *y* = 0.56239 + 0.0004291*x*.

Dietary aTOH levels had no significant impact on dry matter, energy, or protein content of SW‐smolt (Table 6). The VSI and CSI of SW‐smolt were not significantly affected by the dietary aTOH level. However, the HSI of SW‐smolt showed a linear decrease up to a dietary aTOH level of 396 mg aTOH/kg (CI 168–623).

No long‐term effect of the dietary aTOH history on organ indexes, viscerasomatic index (VSI), HSI, CSI, or gonadosomatic index (GSI), were observed in either female or male post‐smolt (Table 7). The CSI and GSI were higher in male fish compared to those in female fish, respectively.

**Table 7:** Viscera‐, hepato‐, cardio‐, and gonadosomatic index (VSI, HSI, CSI, and GSI) of post‐smolt fed graded levels of *α*‐tocopherol during smoltification followed by 6 months of feeding a commercial diet while reared in a common sea cage.

Variable	Females			Males			*p*‐Value		
	VE38	VE260	VE710	VE38	VE260	VE710	Diet	Sex	D × S
VSI	7.4 ± 0.9	6.8 ± 1.1	6.6 ± 1.5	5.7 ± 2.1	6.8 ± 2.5	6.6 ± 1.9	0.95	0.35	0.40
HSI	1.10 ± 0.18	0.94 ± 0.19	0.97 ± 0.28	1.00 ± 0.18	1.01 ± 0.34	1.06 ± 0.19	0.72	0.76	0.56
CSI	0.17 ± 0.02^ab^	0.17 ± 0.02^ab^	0.16 ± 0.02^b^	0.21 ± 0.05^a^	0.16 ± 0.02^ab^	0.18 ± 0.03^ab^	0.23	0.04	0.09
GSI	0.14 ± 0.04	0.10 ± 0.01	0.11 ± 0.01	0.37 ± 0.26	0.28 ± 0.26	0.23 ± 0.20	0.64	0.01	0.74

*Note:* Mean ± standard deviation with *n* = 12 fish for VE38 (*n* = 6 female + 6 male), *n* = 13 for VE260 (*n* = 6 female + 7 male) and *n* = 14 for VE710 (*n* = 5 female + 9 male). Superscript letters indicate significant differences between groups obtained by two‐way ANOVA followed by Tukey HSD.

In FW‐smolt, the whole‐body and liver aTOH levels increased with increasing dietary aTOH levels (Figure 3). Broken‐line regression indicates that the deposition rate of aTOH in the body and liver was higher in FW‐smolt‐fed diets containing aTOH levels above 436 or 405 mg/kg, respectively. Deposition of aTOH in the muscle tissue showed a linear relation to the dietary aTOH levels without saturation. Whole body *γ*‐tocopherol (gTOH) levels remained stable without the impact of dietary aTOH level in FW‐smolt. Nevertheless, liver gTOH significantly increased, while muscle gTOH levels significantly decreased with increasing levels of dietary aTOH, following a linear relationship. Other tocopherol or tocotrienol compounds were largely below the detection limit in liver and muscle tissue (not presented).

**Figure 3:**
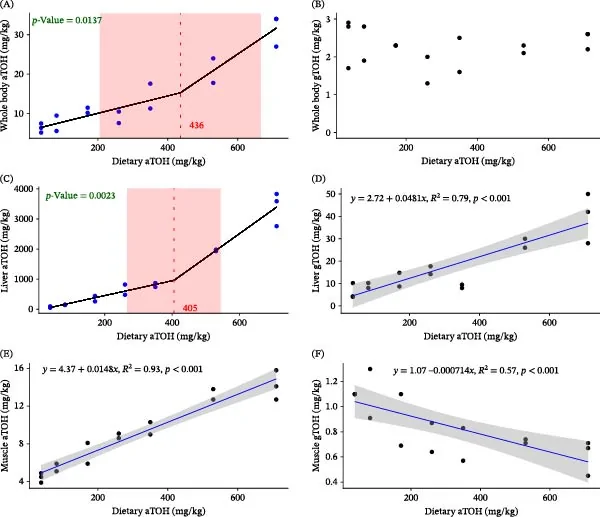
Whole fish (A + B), liver (C + D), and muscle (E + F) tissue *α*‐tocopherol (aTOH; A + C + E) and *γ*‐tocopherol (gTOH; B + D + F) levels in Atlantic salmon FW‐smolt fed graded levels of dietary aTOH. A linear, polynomial, or broken‐line model was fitted to the data, with 95% confidence intervals indicated in red for the broken‐line models and in gray for the others. Equations from the broken‐line model are (A) aTOH ≤ 436: *y* = 5.66063 + 0.02208*x* and aTOH ≥ 436: *y* = −10.771 + 0.05981*x*; (C) aTOH ≤ 405: *y* = −37.683 + 2.459*x* and aTOH ≥ 436: *y* = −2255.31 + 7.991*x*.

In SW‐smolt, whole‐body aTOH showed a linear increase with dietary aTOH levels up to 350 mg aTOH/kg diet, after which whole‐body aTOH levels saturated (Figure 4). Liver aTOH levels showed a 2nd‐order polynomial increase, and gTOH levels a linear increase with increasing dietary aTOH levels in SW‐smolt. The deposition of aTOH in the muscle tissue increased in SW‐smolt with increasing dietary aTOH levels following a linear regression (Figure 3). The gTOH levels in muscle tissue showed no significant effect of dietary aTOH supplementation with SW‐smolt.

**Figure 4:**
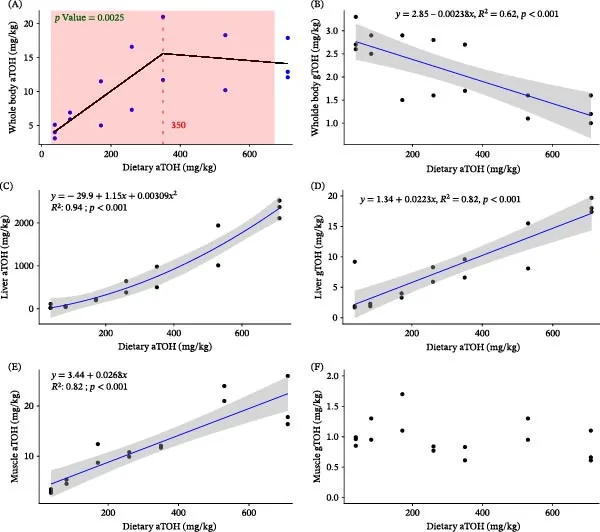
Whole fish (A + B), liver (C + D) and muscle (E + F) tissue *α*‐tocopherol (aTOH; A + C + E) and *γ*‐tocopherol (gTOH; B + D + F) levels in Atlantic salmon 6 weeks after seawater transfer while exposed to temperature and hypoxia challenge in response to graded levels of dietary aTOH. A linear, polynomial, or broken‐line model was fitted to the data, with 95% confidence intervals indicated in red for the broken‐line model and gray for others. The broken‐line equation from (A) aTOH ≤ 350: *y* = 2.704086 + 0.036825*x* and aTOH ≥ 350: *y* = 17.736 − 0.004145*x*.

### 3.4. Tissue Markers

Neither the hematocrit nor the hemoglobin value was affected by dietary aTOH levels in FW‐smolt (Figure 5). The hematocrit value in SW‐smolt peaked at 367 mg aTOH/kg diet, while the hemoglobin value peaked at 450 mg aTOH/kg diet.

**Figure 5:**
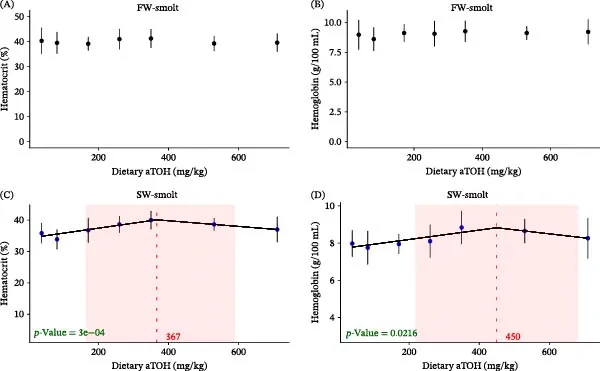
Blood hematocrit (A + C) and hemoglobin (B + D) values in response to graded levels of dietary *α*‐tocopherol in Atlantic salmon fed experimental feeds for 12 weeks during the freshwater phase of the smoltification period (A + B) or 6 weeks after seawater transfer while exposed to temperature and hypoxia challenges (C + D). Broken‐line models with the equations (C) aTOH ≤ 367: *y* = 34.230039 + 0.016016*x* and aTOH ≥ 367: *y* = 43.39 − 0.009028*x*; (D) aTOH ≤ 450: *y* = 7.685428 + 0.002533*x* and aTOH ≥ 450: *y* = 9.797 − 0.002176*x* were fitted to the data, and significant break points are indicated by red dotted lines with 95% confidence intervals marked in red. Mean ± standard deviation with *n* = 12 individual fish per treatment for VE38 and VE710 and *n* = 8 individual fish per treatment for the other diet groups.

Broken‐line regression shows that the liver MDA levels decreased with increasing dietary aTOH levels but stabilized in fish fed diets above 457 mg aTOH/kg diet in FW‐smolt. Similarly, liver MDA levels decreased in SW‐smolt but stabilized when fed dietary aTOH levels above 99 mg/kg (Figure 6).

**Figure 6:**
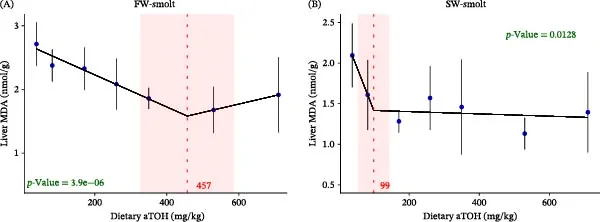
Broken‐line model (break point ± 95% confidence interval) of liver MDA levels in response to graded levels of dietary *α*‐tocoperol in Atlantic salmon fed experimental feeds for 12 weeks during freshwater phase of smoltification period (A) or 6 weeks after seawater transfer while exposed to temperature and hypoxia challenge (B). Mean ± standard deviation with *n* = 12 individual fish per treatment for VE38 and VE710 and *n* = 8 individual fish per treatment for other diet groups. Equations from broken‐line model are (A) aTOH ≤ 457: *y* = 2.7322520 − 0.0025189*x* and aTOH ≥ 457: *y* = 0.993 + 0.0013148*x*; (B) aTOH ≤ 99: *y* = 2.514470 − 0.011061*x* and aTOH ≥ 99: *y* = 1.412 − 0.000140*x*.

In FW‐smolt, muscle astaxanthin and liver selenium levels were not significantly affected by dietary aTOH (Table 8).

**Table 8:** Tissue antioxidant parameters in fish fed graded levels of *α*‐tocopherol.

Variable	VE38	VE82	VE171	VE260	VE350	VE530	VE710	Pooled SD	*p*‐Value			Model fit
									LR	P2	BL	
*FW-smolt*												

AST, muscle	0.75	0.65	0.51	0.56	0.76	0.70	0.74	0.12	0.41	0.38	0.38	NV
Se, liver	2.07	1.75	2.10	1.95	2.25	2.00	1.70	0.33	0.35	0.27	0.43	NV

*SW-smolt*												

AST, muscle	0.64	0.79	0.67	0.80	0.78	0.74	0.93	0.10	0.01	0.03	0.52	*y* = 0.665 + 0.000317*x*
Se, liver	1.83	2.05	1.80	1.85	1.50	1.60	1.70	0.19	0.26	0.31	0.83	NV
Vit C, liver	267	260	285	285	245	285	257	42	0.79	0.87	0.89	NV
GSH/GSSG^1^, liver	439 ± 92^b^	NA	NA	NA	NA	NA	553 ± 157^a^	—	—	—	—	ANOVA, *p* = 0.04

*Note:* Superscript letters indicate significant differences between groups according to one‐way ANOVA (*p* = 0.04) followed by Tukey HSD. AST, astaxanthin (mg/kg); Se, selenium (mg/kg); vit C, vitamin C (mg/kg). Mean with *n* = 3 pooled samples for VE38 and VE710 and *n* = 2 pooled samples for VE82, VE171, VE260, VE350, and VE530.

Abbreviations: BL, broken‐line model; BP, breakpoint; CI, 95% confidence interval; GSH, reduced glutathione; GSSG, oxidized glutathione; LR, linear regression model; ns, not significant; P2, 2nd‐degree polynomial model.

^1^GSSG only measured in SW‐smolt VE38 and VE710 (*n* = 12 fish per treatment with mean ± standard deviation).

In SW‐smolt, liver vitamin C or selenium levels were not significantly affected by the dietary aTOH level (Table 8). Muscle astaxanthin levels showed a linear increase with increasing dietary aTOH levels in SW‐smolt, with 45% higher muscle astaxanthin levels in the fish fed the highest aTOH (VE710) compared to the nonsupplemented diet (VE38). The ratio of GSH to GSSG in the liver tissue was significantly lower in SW‐smolt fed the nonsupplemented diet with 38 mg aTOH/kg compared to SW‐smolt fed the diet containing 710 mg aTOH/kg.

## 4. Discussion

### 4.1. No Dose–Response Effect of Dietary aTOH on Fish Growth During Smoltification

The dietary aTOH levels in the present study, ranging from 38 to 710 mg/kg were able to support normal growth in Atlantic salmon during both the freshwater and seawater phases of smoltification without any observable impact of aTOH levels. This agrees with previous studies in meager (*Argyrosomus regius*), red drum (*Sciaenops ocellatus*), and rainbow trout (*Oncorhynchus mykiss*) where body weight of the fish was not affected by dietary vitamin E supplementation in a dose–response manner [15, 16, 38]. However, body weight has been used to establish aTOH requirements in various fish species, including Atlantic salmon, with optimal dietary levels ranging between 28 and 167 mg/kg in hybrid striped bass (*Morone chrysops* female × *M. saxatilis* male), rohu (*Labeo rohita*) fry, mrigal (*Cirrhinus mrigala*) fry, spotted snakehead (*Channa punctatus*), largemouth bass (*Micropterus salmoide*), and 20 mg/kg, specifically for Atlantic salmon at first feeding [3, 17, 18, 39–41]. The inconsistent effects of aTOH supplementation on growth performance may not only result from species‐specific differences but also from variation in experimental diet formulations. Impaired growth may thereby be considered of low specificity with regard to vitamin E but rather as a general consequence of a nutrient deficiency. Indeed, most requirement studies reporting growth effects were conducted using purified or semipurified diets. Additionally, it has been suggested that aTOH deficiency symptoms may be mitigated in the presence of sufficient vitamin C and selenium [26]. Previous studies investigating vitamin E requirements in fish span a broad range of vitamin C levels ranging from 40 to 5000 mg/kg, while known selenium requirements were typically met. In Atlantic salmon, the vitamin E requirement at first feeding was established using 1188 mg/kg vitamin C [3]. In this context, the 399 mg/kg vitamin C used in the present study is above requirements in salmon but falls within a moderate range, especially considering that a combined supra‐supplementation of vitamins might also suppress growth during smoltification. In a previous study, Atlantic salmon smolt given 430–466 mg/kg vitamin E in combination with 1570–1590 mg/kg vitamin C showed lower growth when compared to fish fed moderate levels of 180 mg vitamin E/kg diet and 230–252 mg vitamin C/kg diet [42]. Besides antioxidants, interactions of vitamin E with dietary levels of other lipid‐soluble vitamins, including vitamin K and A, could similarly be assumed from studies in rodents [43, 44] but are less well characterized to date in fish. Micronutrient levels, other than vitamin E, did not differ between treatments in the present study, and therefore, the low specificity of growth responses may explain why tissue vitamin E levels and biomarkers of target pathways were more sensitive to the applied dietary range of vitamin E than growth.

The dose–response effect of dietary vitamin E on feed intake observed during the freshwater phase of the present study is not commonly reported in fish. For example, research on rainbow trout demonstrated no significant differences in the feed intake of fish [45, 46]. Similarly, in largemouth bass, cosupplementation of graded levels of vitamin E (160–400 mg/kg) and selenium did not significantly impact feed intake in diets with oxidized fish oil [47]. However, a separate study working with lower levels of vitamin E reported reduced feed intake in largemouth bass fed diets containing less than 73 mg/kg [39]. These findings suggest that the effect of vitamin E on feed intake may be limited to low dietary levels and may not be evident at all life stages.

### 4.2. High aTOH Accumulation in Liver Tissue at Excess Supplementation

The response pattern of vitamin E in the body tissue towards increasing levels of dietary vitamin E was similar to those observed in previous studies. Notably, in the present study, whole‐body aTOH levels showed a linear increase in FW‐smolt up to a dietary concentration of 436 mg/kg, consistent with the work of Hamre et al. [6], which utilized graded aTOH levels ranging 48–339 mg/kg. In the present study, the FW‐smolt body aTOH levels did not reach saturation, demonstrating an elevated deposition in fish‐fed diets exceeding 436 mg/kg. However, following transfer to seawater, whole‐body aTOH levels appear to be more tightly regulated in Atlantic salmon, with SW‐smolt displaying saturation when fed 350 mg aTOH/kg. Similarly, Hamre et al. [6] reported a saturation of aTOH in post‐smolts, but at a dietary aTOH level of 140 mg/kg. The discrepancies in saturation levels may reflect differing nutritional requirements arising from variations in experimental conditions, such as the solitary increase of dietary vitamin E in the present study versus the simultaneous increase of multiple antioxidants in the previous study. Additionally, factors such as the life stage and duration of the experiment, which lasted 6 months in the previous trial conducted in sea cages, compared to a sampling period of only 6 weeks post seawater transfer in the present study performed under standardized environmental conditions (mild hypoxia and 14°C temperature), may have also contributed to the observed differences in tissue response.

In the present study, the aTOH levels in muscle tissue followed a linear response, as previously shown for Atlantic salmon [48] as well as rainbow trout [49, 50] and tilapia [51]. The liver aTOH levels followed a 2nd‐degree polynomial increase as previously reported in Atlantic salmon [48] and Caspian trout [52]. At dietary levels below 400 mg/kg, the increase in liver tissue can also be considered linear as previously suggested in red drum‐fed dietary aTOH ranging from 0 to 80 mg/kg [16] and hybrid tilapia fed dietary aTOH ranging from 0 to 201 mg/kg [53]. In the present study, this corresponds to a break point of 405 mg/kg in FW‐smolt. Given that the liver is the primary organ involved in vitamin E metabolism [54], the substantial accumulation at dietary aTOH above 405 mg/kg may indicate an inefficiency in its utilization in excess. Thereby, aTOH is suggested to be the preferred form of vitamin E for retention in the fish liver tissue [55]. Although gTOH can be readily deposited in the muscle tissue of salmon [56], in the present study, the gTOH in whole‐body FW‐smolt and muscle tissue of SW‐smolt showed a linear decrease, while the liver showed a linear increase, supporting the theory of a complex interplay between tocopherol forms in the liver leading to a discriminatory deposition in other tissues [57, 58].

Vitamin E protects lipids from peroxidation but has also been suggested to affect the body’s lipid metabolism [59]. Although no differences in whole‐body lipid levels were observed in this or previous studies on salmonids [3, 53], the dose‐responses on VSI and HSI may point towards tissue‐specific effects. In FW‐smolt, the VSI showed a linear decrease with increasing dietary aTOH levels, while the HSI significantly increased in SW‐smolt‐fed diets above 396 mg/kg. The observed differences between FW‐ and SW‐smolt in the present study might reflect life‐stage‐specific differences with reduced body lipid stores in post‐smolts due to the high energy demand of smoltification. Similarly, in spotted snakehead, the HSI increased when the fish were fed diets up to 140 mg aTOH/kg diet [18]. Further, a histopathological evaluation of liver tissue in meager found that both low and high dietary vitamin E result in increased lipid vacuolization [38]. It was therefore suggested that a healthy lipid metabolism in the liver might be a marker for vitamin E requirements, while it may be disturbed at excess vitamin E. Indeed, TBARS levels as a marker for lipid peroxidation generally decrease under moderate aTOH supplementation in fish; however, in sea bream, it was reported to increase at an excess supplementation of 1000 mg/kg [60].

### 4.3. Dietary aTOH Inhibits Liver MDA Levels With Varying Degrees of Oxidative Stress in FW‐ and SW‐Smolt

Common markers for lipid peroxidation include the determination of MDA and TBARS. These markers are easily responsive to dietary vitamin E and have been commonly used to establish aTOH requirements in fish. Similarly, MDA appears to be a stringent criterion with a clear breakpoint in response to dietary vitamin E in the present study. Most studies measured TBARS in the liver tissue, giving optimal dietary aTOH inclusion levels ranging from 31 to 140 mg/kg [16, 18, 41]. In rainbow trout muscle, MDA levels were reported to have a breakpoint of 100 mg/kg [61], which agrees with the breakpoint obtained in SW‐smolt in the present study at 99 mg/kg. However, in FW‐smolt, breakpoint analysis suggests a requirement of 457 mg/kg, which is closer to that obtained in meager at 451 mg/kg using a muscle TBARS assay [38]. Overall, the breakpoint of 99 mg/kg as observed in SW‐smolt is closer to the aTOH requirement levels previously defined in Atlantic salmon [3], while the breakpoint of 457 mg/kg is closer to those obtained in the present study through tissue aTOH levels. Notably, the daily weight gain and HSI were reduced in SW‐smolt compared to FW‐smolt. As a product of lipid oxidation, liver lipid levels (not measured in the current study) can have a direct impact on MDA formation [62], suggesting that the present observations also reflect life‐stage differences between FW‐ and SW‐smolt, with FW‐smolt experiencing more oxidative stress compared to SW‐smolt. Another factor to be considered is the interaction of vitamin E with other antioxidants. In the present study, liver levels of vitamin C and selenium remained unaffected by dietary aTOH, but SW‐smolt exhibited a significant increase in muscle astaxanthin, with levels up to 45% higher in the highest compared to the lowest aTOH treatment. A similar increase of 8%–14% has been observed by Bjerkeng et al. [63] when using 200 vs 800 mg/kg vitamin E. This suggests that excess vitamin E may exert a sparing effect in the muscle, where astaxanthin is utilized as an antioxidant under oxidative stress [64]. Consequently, dietary aTOH may preserve better fillet quality under challenging conditions [63, 65].

### 4.4. Dietary aTOH Supports Blood Parameters in SW‐Smolt

A decrease in the blood parameters Hgb and Hct is usually considered a clear indication for vitamin E deficiency, posing the risk for anemia in fish [4]. In the present study, no effect of dietary aTOH level on Hgb or Hct could be observed in FW‐smolt, possibly because all diets contained sufficient aTOH to prevent clinical deficiency signs. However, the present results indicate that the optimal dietary aTOH levels to support blood Hct and Hgb values in Atlantic salmon SW‐smolt are 367–450 mg/kg. This is significantly higher than the previously reported levels of 19–140 mg/kg, which were associated with blood parameters in Atlantic salmon and other fish species at different life stages [4, 18, 40, 41]. The differences might be a result of the natural increase in blood parameters during smoltification. The Hct levels in salmon follow a distinct pattern, reaching peak levels of about 40%–45% in fish post seawater transfer [66]. In the present study, the Hct levels were generally lower, reaching 40% only in groups fed dietary aTOH above 367 mg/kg. This might be a result of the environmental conditions of the trial employing mild hypoxia and increased water temperature for fish post seawater transfer, both known to affect blood Hct levels in fish [67].

### 4.5. Low aTOH Affects Cataract Formation and Vertebra Fusion in Atlantic Salmon Post seawater Transfer

Overall, welfare scores in the present study were low across the various life stages, indicating good fish welfare. However, a high prevalence of severe cataract was observed in SW‐smolt, with the highest number of fish with a cataract score above 4 in the group fed the lowest vitamin E diet, indicating that this level was too low to maintain eye health. The etiology of cataract formation is multifactorial (reviewed by Bjerkås et al. [68]), and the role of vitamin E might be linked to protection from oxidative stress that can disrupt lens proteins and membrane lipids. Previous studies investigating the effect of dietary vitamin E in Atlantic salmon have not found effects on cataract development, however, using dietary concentrations above the lowest vitamin E diet used in the present study and lower rearing temperatures [12, 69]. In Waagbø et al. [69], high liver aTOH concentrations were, however, associated with increased cataract formation, which could be related to the increase in cataract prevalence and scores for the high vitamin E group in the present study, although no pro‐oxidative effects were seen in the liver after SW transfer despite high accumulation of aTOH.

Additionally, although the levels of vertebral deformities in this study were low, post‐smolt fish fed the lowest levels of vitamin E during smoltification had a significantly increased risk of developing a complete vertebral fusion (vertebral deformity type 7). In deformity type 7, two (or three) fused vertebrae remodel into an elongated vertebra with a normal amphicoelous structure with two (or three) neural/hemal aches or ribs [70]. Development of type 7 deformity has been suggested to reflect an active repair mechanism for vertebral deformities in Atlantic salmon [71, 72]. This type of deformity may develop in Atlantic salmon pre‐ and post seawater transfer [70] but was only observed in post‐smolt in the present study. Why low vitamin E should induce complete fusions in Atlantic salmon is at present not clear. Fish were unfortunately not radiographed at the end of the feeding period with different feeds. Thus, it is not possible to evaluate if the observed effect is related to a negative effect of low vitamin E and/or a positive and therapeutic effect of the elevated vitamin E when the fish switched to a commercial feed as post‐smolts. Related to the current observation, it is noteworthy that oxidative stress plays a vital role during intervertebral disc degeneration in humans [73] and that vertebra fusion may start with changes in the intervertebral tissue in Atlantic salmon [74–76]. Complete fusions have indeed been induced by exposure to hyperthermic conditions during embryogenesis and juvenile rearing in Atlantic salmon [77]. Further, Atlantic salmon reared at elevated temperatures with developing deformities showed an upregulation of the redox‐sensitive gene *matrix metalloproteinase 13* (*mmp-13*) [78]. These observations possibly reflect an interplay between bone pathologies induced by oxidative stress and active repair mechanisms. Existing literature in mice indicates that vitamin E plays an essential role in bone remodeling, reporting high bone mass formation under low aTOH levels due to decreased bone resorption by osteoclasts [79]. Similar studies on the role of vitamin E on skeletal health in fish are lacking.

## 5. Conclusion

The dietary aTOH levels ranging from 38 to 710 mg/kg were able to support normal growth in Atlantic salmon during smoltification. Fish fed 38 mg/kg showed an elevated prevalence for cataract 6 weeks after seawater transfer and for type 7 vertebra deformity 5 months after transfer to sea cages, suggesting that 82 mg/kg is the minimum dietary aTOH level to avoid welfare issues in farmed Atlantic salmon. An increased deposition of aTOH in the liver tissue above 400 mg/kg was observed. Similarly, liver MDA in FW‐smolt and hematocrit and hemoglobin values in SW‐smolt showed breakpoints at around 367–457 mg/kg, suggesting limited benefits from supplementing above this level and a requirement under the trial conditions of mild hypoxia and elevated temperature post seawater transfer. The linear increase in muscle astaxanthin in response to increasing dietary aTOH further suggests that excess vitamin E may have a sparing effect for antioxidants in the muscle and could improve fillet quality under these challenging conditions.

## Author Contributions

**Pauline Wischhusen**: formal analysis, investigation, data curation, writing – original draft, writing – review and editing, visualization, project administration. **Kristin Hamre and Ingunn Stubhaug**: conceptualization, writing – review and editing. **Per Gunnar Fjelldal**: investigation, resources, writing – review and editing. **Kaja H. Skjærven and Sofie C. Remø**: investigation, writing – review and editing. **Katerina Kousoulaki and Anne-Catrin Adam**: resources, writing – review and editing. **Antony Jesu Prabhu Philip**: conceptualization, writing – review and editing, project administration, funding acquisition. **Nini H. Sissener**: conceptualization, methodology, investigation, writing – review and editing, supervision, project administration, funding acquisition.

## Funding

This work received funding from the Fiskeri‐ og havbruksnæringens forskningsfond (Norwegian Seafood Research Fund, FHF) through the project “Revitalise – Requirement of vitamin and trace minerals for Atlantic salmon health” (Grant 901834). The funders had no role in the study design, data collection and analysis, decision to publish, or preparation of the manuscript. Open Access funding provided by the Havforskningsinstituttet.

## Disclosure

All authors have read and approved the manuscript.

## Conflicts of Interest

The authors declare no conflicts of interest.

## Supporting Information

Additional supporting information can be found online in the Supporting Information section.

## Supporting information


**Supporting Information** Table S1: Model selection parameters for biometric measures in fish fed graded levels of *α*‐tocopherol. Table S2: Model selection parameters for proximate composition and organ indexes of fish fed graded levels of *α*‐tocopherol. Table S3. Model selection parameters for tissue antioxidant parameters in fish fed graded levels of *α*‐tocopherol.

## Data Availability

The data that support the findings of this study are available from the corresponding author upon reasonable request.
