# Supplementary material for: Vitamin E Requirement in Practical Feed for Farmed Atlantic Salmon (Salmo salar) During Smoltification and Its Long‐Term Impact on Fish Welfare
Source: Aquac Nutr. 2026 Nov 1;2026:8100107. doi: 10.1155/anu/8100107 (PMC13724154; doi:10.1155/anu/8100107)
Supplement: Supplementary file 1 [file ANU-2026-8100107-s001.pdf]

Table S1. Model selection parameters for biometric measures in fish fed graded levels of α-tocopherol

	**Linear regression**			**2nd degree polynom**			**Broken-line regression**			**Model selection**
	AIC	R^2	*p*	AIC	R^2	*p*	AIC	R^2	*p*	
FW-smolt										
BW	2100	0.00	0.58	2101	0.00	0.44	2102	0.00	0.64	NV
K	-249	0.00	0.95	-247	-0.01	0.99	-245	-0.01	0.83	NV
DWG	1.63	-0.05	0.62	3.28	-0.11	0.77	2.84	-0.03	0.68	NV
SGR	-28.6	-0.05	0.61	-26.9	-0.11	0.78	-27.4	-0.03	0.68	NV
TGC	-13.0	-0.05	0.59	-11.7	-0.08	0.66	-9.22	-0.21	0.67	NV
FCR	-18.6	0.01	0.29	-19.8	0.13	0.16	-23.4	0.34	0.17	NV
FI	-27.0	0.06	0.18	-30.3	0.28	0.05	-45.6	0.73	<0.01	BP 82; CI 63-101
										aTOH ≤ 82: y=1.10108 + 0.005154x
										aTOH ≥ 82: y=1.4335 + -0.000066x
SW-smolt										
BW	15356	0.00	0.46	15358	0.00	0.60	15359	0.00	0.68	NV
K	-1790	0.00	0.24	-1791	0.00	0.08	-1789	0.00	0.21	NV
DWG	7.48	-0.06	0.78	7.20	0.00	0.38	6.41	0.09	0.58	NV
SGR	-6.87	-0.06	0.78	-7.35	0.02	0.35	-8.00	0.10	0.57	NV
TGC	5.33	-0.07	0.82	4.78	0.02	0.35	4.29	0.09	0.58	NV
FCR	-8.84	-0.07	0.94	-9.37	0.01	0.36	-10.7	0.13	0.55	NV
FI	1.56	-0.06	0.71	2.76	-0.09	0.67	1.19	0.06	0.24	NV
Post-smolt										
BW	5064	0.00	0.91	5066	0.00	0.72	5068	0.00	0.64	NV
K	-247	0.00	0.93	-248	0.00	0.19	-248	0.01	0.31	NV
SGR	140	0.00	0.99	142	0.00	0.99	144	0.01	0.67	NV

BW, final body weight; K, condition factor; DWG, daily weight gain; SGR, specific growth rate; TGC, thermal growth rate; FCR, feed conversion ratio; FI, feed intake

Table S2. Model selection parameters for proximate composition and organ indexes of fish fed graded levels of α -tocopherol.

	Linear regression			2nd degree polynom			Broken-line regression			Model selection
	AIC	R^2	*p*	AIC	R^2	*p*	AIC	R^2	*p*	
FW-smolt										
DM	33.5	-0.02	0.40	34.4	-0.02	0.45	33.3	0.09	0.60	NV
Energy	222	-0.06	0.67	223	-0.13	0.87	224	-0.11	0.75	NV
Lipid	22.2	-0.06	0.76	23.6	-0.10	0.74	23.9	-0.08	0.56	NV
Protein	27.2	-0.06	0.68	29.1	-0.14	0.91	30.6	-0.19	0.87	NV
VSI	225	0.07	0.02	227	0.06	0.05	228	0.06	0.18	y=7.8-0.00167x
HSI	-7.47	-0.02	0.82	-6.13	-0.02	0.71	-5.12	-0.02	0.52	NV
CSI	-350	0.02	0.11	-352	0.07	0.04	-354	0.10	0.05	BP530; CI372-688
										aTOH ≤ 530: y=0.1324 + 0.00003793x
										aTOH ≥ 530: y=0.1716 -0.00007437x
SW-smolt										
DM	26.5	0.01	0.29	28.2	-0.04	0.52	30.0	-0.12	0.91	NV
Energy	217	-0.02	0.42	219	-0.05	0.56	220	-0.13	0.92	NV
Lipid	19.9	0.00	0.36	18.4	0.13	0.16	19.6	0.10	0.24	NV
Protein	27.0	0.05	0.20	28.7	0.00	0.39	30.0	-0.04	0.55	NV
Vitamin C	164	-0.06	0.79	166	-0.13	0.87	168	-0.21	0.89	NV
VSI	350	0.01	0.17	352	0.00	0.39	353	-0.01	0.75	NV
HSI	-83.1	-0.01	0.81	-87.3	0.06	0.05	-86.7	0.07	0.03	BP395; CI168-623
										aTOH ≤ 396: y=0.8623329-0.0003290x
										aTOH ≥ 396: y=0.5623520 -0.0004291x
CSI	-260	-0.01	0.54	-259	-0.01	0.44	-257	-0.02	0.40	NV

DM, dry matter; VSI, viscera-somatic index; HSI, hepato-somatic index; CSI, cardio-somatic index

Table S3. Model selection parameters for tissue antioxidant parameters in fish fed graded levels of α-tocopherol

	**Linear regression**			**2nd degree polynom**			**Broken-line regression**			**Model selection**
	AIC	R^2	*p*	AIC	R^2	*p*	AIC	R^2	*p*	
FW-smolt										
AST, muscle	-16.0	-0.02	0.41	-15.6	0.01	0.38	-17.8	0.17	0.38	NV
Se, liver	12.9	0.00	0.35	12.7	0.05	0.27	14.2	0.01	0.43	NV
SW-smolt										
AST, muscle	-25.1	0.37	0.01	-23.3	0.33	0.03	-22.7	0.34	0.52	y=0.665 + 0.000317x
Se, liver	0.65	0.03	0.26	1.30	0.04	0.31	2.27	0.02	0.83	NV
Vitamin C,liver	164	-0.06	0.79	166	-0.13	0.87	168	-0.21	0.89	NV

AST, astaxanthin; Se, selenium.
